# Microbial Ecology of Methanotrophy in Streams Along a Gradient of CH_4_ Availability

**DOI:** 10.3389/fmicb.2020.00771

**Published:** 2020-05-14

**Authors:** Alexandre Bagnoud, Paraskevi Pramateftaki, Matthew J. Bogard, Tom J. Battin, Hannes Peter

**Affiliations:** ^1^Stream Biofilm and Ecosystem Research Laboratory, School of Architecture, Civil and Environmental Engineering, École Polytechnique Fédérale de Lausanne, Lausanne, Switzerland; ^2^Groupe de recherche interuniversitaire en limnologie, Département des sciences biologiques, Université du Québec à Montréal, Montréal, QC, Canada

**Keywords:** *Methylococcaceae*, *Crenothrix*, Michaelis–Menten, enterotypes, *pmoA*

## Abstract

Despite the recognition of streams and rivers as sources of methane (CH_4_) to the atmosphere, the role of CH_4_ oxidation (MOX) in these ecosystems remains poorly understood to date. Here, we measured the kinetics of MOX in stream sediments of 14 sites to resolve the ecophysiology of CH_4_ oxidizing bacteria (MOB) communities. The streams cover a gradient of land cover and associated physicochemical parameter and differed in stream- and porewater CH_4_ concentrations. Michealis–Menten kinetic parameter of MOX, maximum reaction velocity (*V*_*max*_), and CH_4_ concentration at half *V*_*max*_ (*K*_*S*_) increased with CH_4_ supply. *K*_*S*_ values in the micromolar range matched the CH_4_ concentrations measured in shallow stream sediments and indicate that MOX is mostly driven by low-affinity MOB. 16S rRNA gene sequencing identified MOB classified as *Methylococcaceae* and particularly *Crenothrix*. Their relative abundance correlated with *pmoA* gene counts and MOX rates, underscoring their pivotal role as CH_4_ oxidizers in stream sediments. Building on the concept of enterotypes, we identify two distinct groups of co-occurring MOB. While there was no taxonomic difference among the members of each cluster, one cluster contained abundant and common MOB, whereas the other cluster contained rare operational taxonomic units (OTUs) specific to a subset of streams. These integrated analyses of changes in MOB community structure, gene abundance, and the corresponding ecosystem process contribute to a better understanding of the distal controls on MOX in streams.

## Introduction

In streambeds, microorganisms organized in surface-attached and matrix-enclosed biofilms drive biogeochemical processes that regulate the outgassing of carbon dioxide (CO_2_), methane (CH_4_), and nitrous oxide (N_2_O) (Battin et al., 2016). While the study of stream CO_2_ biogeochemistry has received considerable attention over the last decade, the biogeochemistry of CH_4_ (Crawford et al., 2014; Trimmer et al., 2015; Stanley et al., 2016) and specifically the microbial ecology of aerobic CH_4_ oxidizing bacteria (MOB) in streams remains poorly understood (Trimmer et al., 2015, 2010; Stanley et al., 2016). Streams outgas substantial amounts of CH_4_ originating from groundwater and methanogenesis within the streambed (Chaudhary et al., 2014; Stanley et al., 2016). Globally, streams and rivers may emit 26.8 Tg CH_4_ per year, which is similar to the combined contribution of other natural CH_4_ sources such as wildfires, termites, CH_4_ hydrates, and permafrost (Stanley et al., 2016). The presence of wetlands and human activity in stream catchments are positively related to streamwater CH_4_ concentrations and CH_4_ evasion fluxes (Chaudhary et al., 2014; Stanley et al., 2016). Land cover is linked to streamwater CH_4_ concentration, as the excess delivery of organic matter and nutrients leads to oxygen depletion and thus stimulates anaerobic methanogenesis.

MOB constitute the main microbial processors of CH_4_ at oxic/anoxic interfaces in stream sediments (Trimmer et al., 2010, 2015; Buriánková et al., 2012; Shelley et al., 2014, 2015) where MOB can account for up to 30% of prokaryotic cell numbers (Buriánková et al., 2012). CH_4_ oxidation (MOX) can be an important ecosystem process, equivalent to up to 46% of net photosynthetic production in chalk streams (Trimmer et al., 2010; Shelley et al., 2014) and reducing CH_4_ evasion fluxes by 70% (Zaiss et al., 1981; de Angelis and Scranton, 1993). Among MOB, low-affinity CH_4_ oxidizers with substrate affinity constants K_*S*_ [i.e., the substrate concentration for which the MOX rate is half the maximum reaction velocity (*V*_*max*_)] in the micromolar range are adapted to elevated CH_4_ concentrations typical for hot spots of methanogenesis. In contrast, high-affinity oxidizers with *K*_*S*_ values in the nanomolar range are adapted to atmospheric CH_4_ concentrations (Bender and Conrad, 1992). Independent of their kinetic affinity, known MOB are distributed across *Methylocystaceae, Beijerinckiaceae* (both α-Proteobacteria, or type II MOB), *Methylococcaceae, Methylothermaceae* (both γ-Proteobacteria, or type I MOB), and *Methylacidiphilaceae* (Verrucomicrobia) (Knief, 2015). Proteobacterial MOB dominate methanotrophic communities in most environments and were previously associated with two life strategies (Ho et al., 2013): γ-proteobacterial MOB are generally responsive to changes in CH_4_ concentrations by rapidly inducing *pmoA* gene expression (coding for a subunit of the particulate CH_4_ monooxygenase). Although numerically not abundant in many environments, they functionally dominate MOX when CH_4_ concentrations are high. Recent studies revealed the importance of *Crenothrix*, a filamentous type I MOB in lakes (Oswald et al., 2017) and caves (Karwautz et al., 2018). In contrast, α-proteobacterial MOB typically feature more stable populations with less responsiveness to fluctuating CH_4_ concentrations (Ho et al., 2013).

The aim of this study was to link MOX kinetics to MOB community structure and genetic potential of stream sedimentary communities. Experimenting with stream sediments sampled across a large environmental gradient (including alpine, natural, urban, and agriculture-dominated catchments), we hypothesized that the kinetics of MOX depend on CH_4_ supply, which we expected to predictably vary with land use. By characterizing changes in the MOB community (membership and overall structure) and measuring both the gene and corresponding ecosystem processes across environmental gradients, we aimed to better understand the distal controls on MOX in streams. Elucidating the changes in MOX and MOB communities is critical, given the apparent relationship between land use, nutrient, and CH_4_ dynamics in streams (Stanley et al., 2016) and recent predictions that eutrophication will continue to increase during the 21st century (Sinha et al., 2017).

## Materials and Methods

### Study Sites and Sampling

We sampled 14 sites (located between 378 and 1,189 m above sea level) of nine streams in Switzerland between March and July 2016. Using QGIS, we derived the percent land cover for each of the catchments using European Environment Agency’s 2018 Corine Land Cover (CLC, Version 20) database. Dominant land cover types included non-irrigated arable land, mixed forest, coniferous forest, natural grasslands, bare rock, discontinuous urban fabric, and land principally occupied by agriculture with significant areas of natural vegetation and transitional woodland-shrub (Supplementary Table S1).

At each site, we collected triplicate benthic sediment samples (down to 5 cm depth) from which we retained the sandy fraction (<2 mm). Sediment subsamples for DNA extraction were immediately transferred to LifeGuard Soil Preservation Solution (Mobio) and stored at −80°C until processing. Subsamples for flow cytometric cell counting were immediately fixed with 2.5% formaldehyde (final concentration) and kept cool (4°C). Sediments were transported cooled to the laboratory for kinetic analyses (see below). Porewater was sampled using a 1.5-cm diameter piezometer connected to a syringe. For the determination of dissolved CH_4_ and CO_2_, we collected 30 ml stream- and porewater into 60 ml syringes flushed with N_2_ and containing 0.05 g of NaN_3_ to stop biological activity. Stream- and porewater dissolved oxygen (DO) was directly measured with an LDO probe (Hach) equipped with a flow-through cell connected to the piezometer. Another set of stream- and porewater samples were filtered [0.2 μm polytetrafluoroethylene (PTFE) filters] and treated with 1 M HCl for the measurement of Fe(II) or 5% (w/v) zinc acetate for S(-II) measurements. Stream- and porewater samples for the determination of dissolved organic carbon (DOC) concentration were filtered through pre-combusted (450°C for 4 h) GF/F filters (Whatman).

### Geochemical Analyses

Anions and cations were measured by ion chromatography (DX-3000, Dionex) with an IonPac AS11-HC column (anions) and an IonPac CS16 Cation-Exchange column (cations). S(-II) and Fe(II) were measured photometrically (Cline, 1969; Stookey, 1970). DOC concentration was measured using a Sievers M5310C TOC Analyzer. CO_2_ and CH_4_ concentrations in the streamwater and porewater were measured with the headspace equilibration method and using cavity ring-down spectroscopy (Picarro G2201-I). First, we added ultrapure N_2_ (30 ml) to the syringes containing the sample, which we shook (1 min) to allow gases to equilibrate with the headspace. The gases were then transferred to a gas-tight glass syringe (N_2_-flushed) and diluted to a final volume of 50 ml with N_2_. Partial pressures of CO_2_ and CH_4_ in the headspace samples were converted to gas concentration in the water samples as described previously (Bagnoud et al., 2016). The surface area of dry sediments was measured using the Brunauer–Emmett–Teller method (Gemini 2375 V4.01; Micromeritics). All rates and microbial parameters were normalized to sediment surface area.

### Methane Oxidation Kinetics

We derived MOX kinetics from laboratory incubations in round, 11 cm in diameter and 8 cm in height Plexiglas chambers (volume: 760 cm^3^). The chambers featured in- and outlet valves at the lid and were sealed airtight using screws and a rubber seal. Prior to the experiments, the respective streamwater was equilibrated with air amended with 1% CH_4_ (Segers, 1998). For each site, triplicated incubations were set up with approximately 25 g of sediment (wet weight), which covered the bottom of the chambers with a thin layer. The chambers were then filled headspace-free using CH_4_-amended streamwater. To avoid diffusion limitation, magnetic stirring bars in the center of the incubation chambers ensured continuous mixing of the water within the chambers (sediment was initially pushed toward the sides of the chambers but then remained undisturbed). Triplicated incubations without sediment for each site were used to estimate the contribution of streamwater microbial communities to MOX in the incubations. The incubations were performed in the dark and at 20°C ± 2°C. Concentrations of dissolved O_2_ (DO), CH_4_, and CO_2_ were monitored twice per day in each chamber over 3–5 days, depending on MOX rates. DO was measured using optical sensors (SP-PSt3-NAU, Presens, Germany) mounted to the inside of the chambers. The optical DO reading was obtained through the Plexiglass. For the measurement of CH_4_ and CO_2_, 30 ml of water was withdrawn from the chambers and measured using the headspace equilibration method and cavity ring-down spectroscopy as described above. The removed water was simultaneously replaced with unamended streamwater (equilibrated with air), and we accounted for dilution effects by measuring CH_4_, CO_2_, and O_2_ concentrations therein.

Kinetic parameters of MOX were modeled based on Michaelis–Menten:

(1) $$V=V_{m\,a\,x}\cdot S/\left(K_{s}+S\right),$$

where *V* is the MOX velocity (or rate), *V*_*max*_ the maximum reaction velocity, *S* the CH_4_ concentration, and *K*_*s*_ the concentration for which *V* = *V*_*max*_/2 (half-saturation constant).

### 16S rDNA Amplicon Sequencing and Bioinformatic Analyses

DNA was extracted from sediments collected in the field using the Fast DNA spin kit (MP Bio). 16S rRNA genes were amplified using the 515F/806R primers (Caporaso et al., 2011) overhanged with adapter sequences according to the MiSeq manufacturer’s instructions, using an annealing temperature of 50°C. Library preparation and sequencing on an Illumina MiSeq platform (250 bp paired-end) was performed by the LGTF sequencing facility (University of Lausanne).

Reads were analyzed using the UPARSE pipeline (Edgar, 2013), including the removal of unique reads, reads with an expected error >1, and chimeric sequences. Reads were clustered to operational taxonomic units (OTUs) at 97% sequence identity. The RDP Classifier v.1.12 was used to assign taxonomy to OTUs with a confidence threshold of 0.8 (Wang et al., 2007). Raw and representative OTU sequences were deposited to NCBI SRA and NCBI Genbank, respectively. Both datasets can be accessed under BioSample numbers SAMN07174972-SAMN07174985.

A reference tree for *Methylococcacea* was constructed using published sequences (Bowman, 2015) using IQ-TREE v.1.5.3 (Nguyen et al., 2015). References were aligned with MAFFT v.7.309 (Katoh and Standley, 2013). Sequences of *Methylococcaceae* OTUs were added to the reference alignment using MAFFT. RAxML v.8.2.9 (Stamatakis, 2014) was used to compute the evolutionary placements of the OTUs on the tree.

### Quantitative PCR

We used the primer pair A189 F/Mb661 R (Holmes et al., 1995; Costello and Lidstrom, 1999) for the quantification of the *pmoA* gene. Reactions were performed in duplicate on a MIC instrument (Bio Molecular Systems) using the SensiFAST SYBR No-ROX kit (Bioline) containing 1 × PCR buffer, 500 nM of each primer, 0.1 ng μl^−1^ bovine serum albumin (BSA; Invitrogen) and 2.5 μl template DNA. Various five-fold dilutions of the DNA extracts (up to 1:125 or 1:625 in water) were examined to assess the efficiency of the amplification assay for each sample. For every sample, the dilution resulting in a ΔCq closer to the theoretical value for five-fold dilutions (2.3 cycles) was used for further analysis. After an initial denaturation at 94°C for 5 min, 45 cycles were performed at 95°C for 15 s, 60°C for 20 s, and 72°C for 30 s. Data acquisition was performed at 82°C for 5 s to avoid fluorescent signal from primer dimers. Melt curve analysis was performed from 72 to 95°C in 0.3°C s^−1^ increments. Agarose gel electrophoresis was used in addition to verify the formation of expected PCR product. Reactions containing all reagents except template DNA were included as negative controls. Amplification efficiency of standard curves ranged from 91 to 95% (R^2^ > 0.99), while corresponding amplification efficiencies of samples showed only minor inhibition of the amplification reaction in a few of them (amplification efficiency 84 to 110% and R^2^ > 0.99). A minimum sensitivity of 10 target molecules per reaction was achieved.

### Bacterial Abundance

Bacterial cells associated with sediments were enumerated using flow cytometry (Novocyte, Acea) (Besemer et al., 2009). Cells were detached and separated from the sediment by shaking (1 h) and sonication (1 min; 1-Hz pulses; 14% amplitude) in pyrophosphate solution (0.025 mM). Samples were diluted (1:10) with MilliQ-water, stained with SYTO13 (Molecular Probes) (25 μM) and identified in plots of fluorescence at 520 nm and side scatter of the 488-nm laser. Volumetric counts, corrected for dilution with fixative and dye, were normalized to sediment surface.

### Statistical Analyses

We used principal component analysis (PCA) to characterize the gradient of streams sampled. For this, we used the normalized concentrations of streamwater NO_3_^–^, SO_4_^2^^–^, Cl^–^, and Ca^2^^+^ and DOC, pH, temperature, and altitude of each site as well as the percent coverage of the major land cover categories (Supplementary Table S1). Next, we fitted stream- and porewater CH_4_ concentrations onto the PCA using the function *envfit* implemented in the R package *vegan* (Oksanen et al., 2017). Pearson’s correlation analyses, linear models, and non-metric multidimensional scaling (nMDS) ordinations were also computed using R. A generalized additive model (function *ordisurf*) was used to fit *pmoA* copy numbers to an nMDS based on Bray–Curtis dissimilarities using the R package *vegan*. In analogy to the identification of community types (Gonze et al., 2017) for which samples are grouped by community composition and form distinct clusters (i.e., enterotypes in the gut microbiome) (Costea et al., 2018), we investigated the contribution of MOB to the microbial composition landscape. For this, we excluded the most oligotrophic sample Adn-1, which contained only a single methanotrophic OTU. Next, we visualized the result of the nMDS ordination as the density landscape of OTUs scores. We then highlighted the location of methanotrophic OTUs in this community composition landscape and identified two distinct clusters of MOB OTUs using k-mean clustering. Finally, we extracted non-MOB OTUs that co-cluster with MOB clusters in the community composition landscape and assessed whether taxonomic groups were relatively overrepresented. We interpret the relative overrepresentation of a taxonomic group associated with the MOB clusters as signs of a positive association of these co-occurring groups.

## Results

### Geochemical Characterization of Streams

**FIGURE 1:**
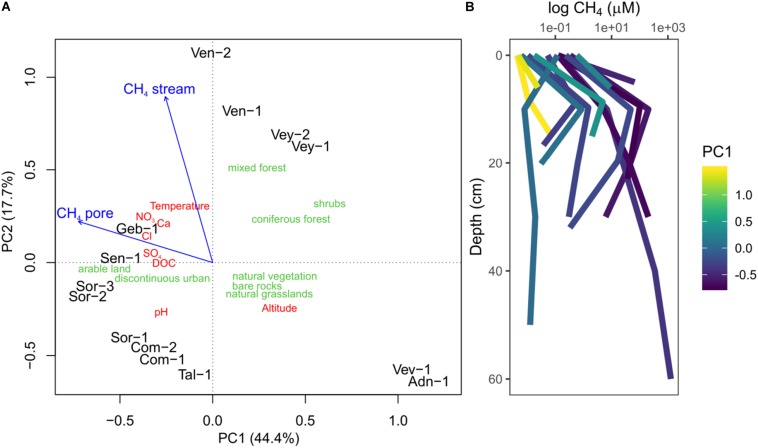
Principal component analysis (PCA) using stream environmental characteristics (red) and land cover type (green) to cluster sampling sites **(A)**. The sampling sites group into three categories: sites at high altitude dominated by bare rock and natural vegetation (Vev-1, And-1), sites with substantial areas of the catchment covered by forests (Ven-2, Ven-1, Vey-2, Vey-1), and sites dominated by arable land and urban areas (Geb-1, Sen-1, Sor-1, Sor-2, Sor-3, Com-1, Com-2, Tal-1). Stream- and porewater methane (CH_4_) concentrations were fitted onto the ordination (blue arrows) and were elevated in sites with large areas of the catchment dominated by arable land and forests. Concentration profiles of CH_4_ measured in streambeds **(B)** color coded according to the site scores on the first PC (PC1). Measurements at 0 cm represent streamwater. Shown are averages of replicated measurements at each depth. Note the peak in CH_4_ concentration at approximately 10 cm depth in most sites.

The 14 sites covered a wide gradient of environmental conditions (Supplementary Table S2 and Figure 1A). The PCA revealed three classes of sites: catchments of Vev-1 and Adn-1 were characterized by natural vegetation, mainly grasslands and bare rocks. These sites were located at high altitude; streamwater temperature and the concentration of ions and DOC were low. Vey-1, Vey-2, Ven-1, and Ven-2 formed another group of samples, with catchments covered to approximately 30% by mixed and coniferous forests and elevated concentrations of ions and DOC in the streamwater. Finally, Geb-1, Sen-1, Sor-1, Sor-2, Sor-3, Com-1, Com-2, and Tal-1 formed another group of samples with arable land and urban regions dominating the catchments. They featured elevated ion and DOC concentrations and higher temperatures as compared to the sites at high altitude. However, compared to the sites dominated by forests, these sites were characterized by elevated pH. Fitting stream and shallow porewater CH_4_ concentrations to the ordination showed that sites at elevated altitude were generally characterized by low CH_4_ concentrations in both stream- and porewater. The group of samples with substantial areas covered by forests had elevated CH_4_ concentration in the streamwater, whereas the sites dominated by agriculture and urban areas were characterized by high porewater CH_4_ concentrations.

In these 14 streams, CH_4_ concentrations were higher in the porewater (average ± standard deviation; 77.75 ± 234.75 μM) than in the streamwater (0.134 ± 0.159 μM) (Figure 1B), except for stream Ven-1. Porewater CH_4_ concentrations often peaked around a depth of 10 cm (Figure 1B and Supplementary Figure S1). Concomitantly with CH_4_, CO_2_ concentration increased on average from 90 ± 78 μM in the streamwater to 541 ± 445 μM in the porewater, while porewater DO concentration decreased from 322 ± 44 to 95 ± 104 μM with depth. Porewater in some streams (Com-2, Sen-1, Sor-3, Ven-2) became anoxic below 10-cm depth (Supplementary Figure S1). Porewater Fe(II) and S(-II) concentrations ranged from 0 to 27 μM and 0 to 4 μM, respectively, and were only above detection limit (0.3 μM) in streams with elevated nitrate concentrations (e.g., Geb-1, Sen-1, Sor-1, Sor-2, Sor-3; Supplementary Figure S1). Concentrations of Fe(II) and NH_4_^+^ (ranging from 0 to 278 μM) followed patterns similar to CH_4_ in the streambeds. Porewater concentrations of SO_4_^2^^–^ and NO_3_^–^ ranged from 70 to 458 μM and from <1 to 402 μM, respectively, and typically decreased with streambed depth (Supplementary Figure S1). Streamwater CH_4_ concentration and the ratio of CH_4_:CO_2_, a proxy for *in situ* CH_4_ production (Stanley et al., 2016), were higher in streams with elevated NO_3_^–^ concentrations (Supplementary Figure S2). Unlike the other streams, Vey-2 drains a wetland with low-redox environments releasing high amounts of CH_4_ but low amounts of nitrate (Supplementary Table S1). Phosphate concentrations were below the detection limit (5 μM) for all measured samples.

### Kinetics of Methane Oxidation in Sedimentary Biofilms

**FIGURE 2:**
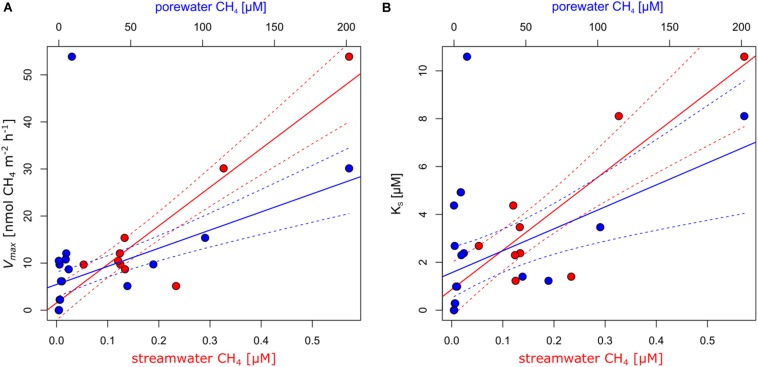
Methane (CH_4_) oxidation (MOX) kinetics constants maximum reaction velocity [*V*_*max*_; **(A)**] and concentration at half *V*_*max*_ [*K*_*S*_; **(B)**] were significantly related to streamwater (red) and porewater (blue) CH_4_ concentrations. The lines represent linear regression models, and the dashed lines represent the respective 95% confidence interval.

Contrasting incubations with and without sediments revealed that MOX was primarily associated with sediments (Supplementary Figures S3–S5). The dynamics of MOX of incubations with sediments were well captured by Michaelis–Menten kinetics (Supplementary Figures S6). Overall, *V*_*max*_ ranged between 0 and 53.9 nmol m^–^^2^ h^−1^, and *K*_*S*_ ranged between 0 and 10.6 μM. MOX was undetectable in the two alpine sites (*V*_*max*_ = 0 and *K*_*S*_ = 0). Average *V*_*max*_ and *K*_*S*_ in sites dominated by forests reached 20.1 ± 22.7 nmol m^–^^2^ h^−1^ (average ± standard deviation) and 5.3 ± 3.8 μM. Highest *V*_*max*_ and *K*_*S*_ were detected in Vey-2, the site draining a wetland. Excluding this site reduced average *V*_*max*_ to 8.8 ± 3.2 nmol m^–^^2^ h^−1^ and *K*_*S*_ to 3.6 ± 1.9 μM for this group of samples. Streams dominated by agriculture and urban areas reached an average *V*_*max*_ of 11.7 ± 8.4 nmol m^–^^2^ h^−1^ and *K*_*S*_ of 2.7 ± 2.4. Both *V*_*max*_ and *K*_*S*_ reflected the CH_4_ supply of the respective streams (Figure 2). Excluding Vey-2, *V*_*max*_ was positively related to streamwater (*R*^2^ = 0.92, *p* < 0.01) and porewater (*R*^2^ = 0.69, *p* < 0.01) CH_4_ concentrations. Similarly, *K*_*S*_ was also positively related to stream- and porewater CH_4_ concentrations (*R*^2^ = 0.84, *p* < 0.01 and *R*^2^ = 0.45, *p* = 0.01, respectively) when excluding Vey-2. Excluding site Ven-1, which had a very low porewater CH_4_ concentration, *K*_*S*_ values reached on average 88.1% of the measured porewater CH_4_ concentrations, however, with considerable variation (range: 4–533%). Given that CH_4_ concentrations experienced by MOB are in the range of the *K*_*S*_ values, putative *in situ* MOX rates estimated by the Michealis–Menten model (Eq. 1) ranged between 0 and 27 nmol CH_4_ m^–^^2^ h^−1^ (Supplementary Table S3). Assuming that 50% of the oxidized CH_4_ was used for biomass production (Trimmer et al., 2015), these putative *in situ* MOX rates translate into carbon fixation rates ranging from 0 and 13.5 nmol C m^–^^2^ h^−1^ (Supplementary Table S3).

### Relating Methane Oxidizing Bacteria to Biofilm Community Structure

We measured *pmoA* gene counts to quantify the abundance of MOB and their contribution to total bacterial abundance and MOX. Total cell abundance ranged on average from 4.6 × 10^6^ to 3.6 × 10^8^ cells m^–^^2^ (Supplementary Table S3). qPCR revealed that the number of cells harboring a *pmoA* coding gene ranged from below detection limit to 3.8 × 10^6^ cells m^–^^2^, with an average of 8.7 ± 9.89 × 10^5^ cells m^–^^2^ (Supplementary Table S3). Thus, *pmoA*-harboring cells contributed on average 0.7 ± 0.69% to total cell abundance, with a maximum contribution of 2.65% in the sediment of Sen-1.

**FIGURE 3:**
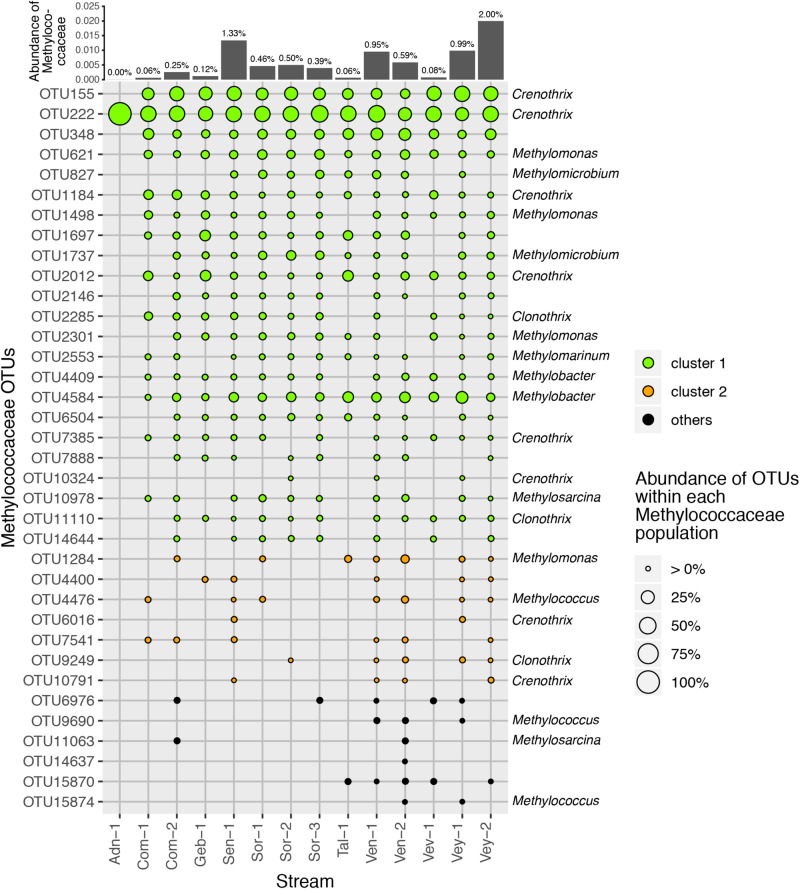
Operational taxonomic units (OTUs) classified as *Methylococcaceae* and particularly *Crenothrix* were present in all samples. The relative abundance of each OTU is represented by the symbol size, the color of each symbol reflects cluster affiliation (see Figure 5). The relative abundance of *Methylococcaceae* to the total microbial community is indicated for each sample at the top. The genus affiliation (if classified) for each OTU is indicated to the right side of the plot.

**FIGURE 4:**
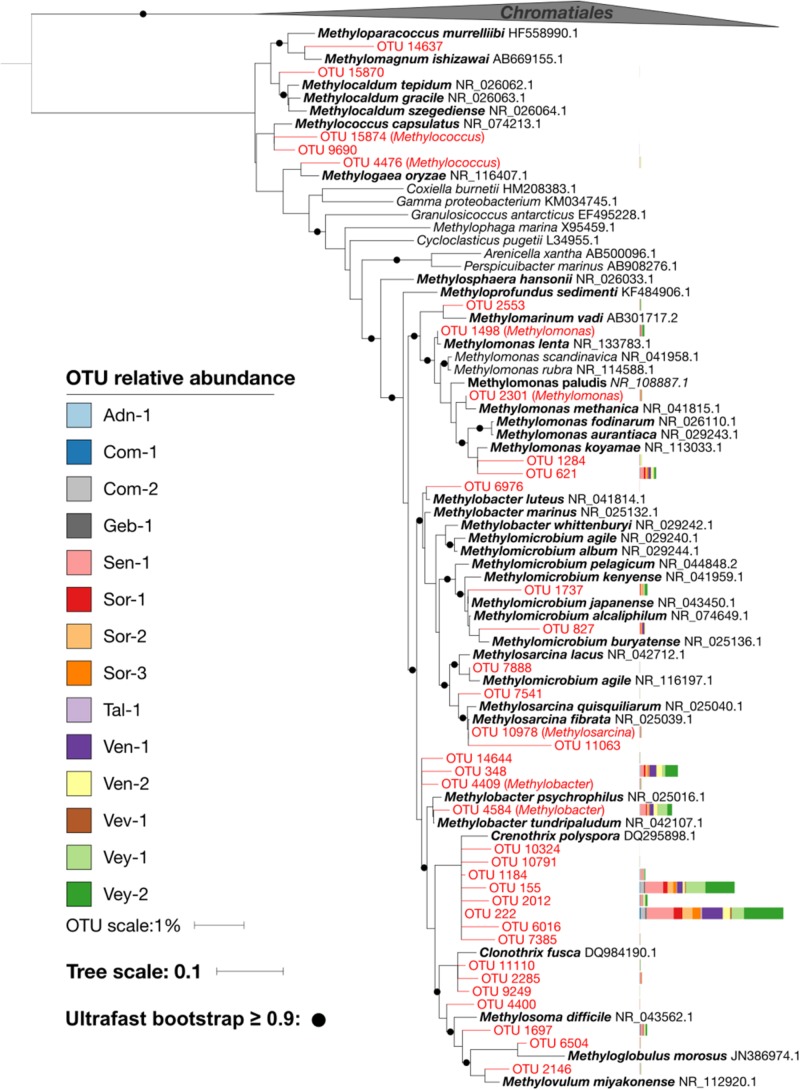
Placement of *Methylococcaceae* operational taxonomic units (OTUs) (red) in a reference phylogenetic tree. Two *Chromatiales* sequences were used as outgroup. Bootstrap values greater than 0.9 are highlighted with black circles. The relative abundance of OTUs in each sample is represented as stacked bars. The majority of and the most abundant *Methylococcaceae* OTUs cluster with *Crenothrix polyspora*.

16S rRNA gene amplicon sequencing resulted in 3.22 million reads that clustered into 16,364 OTUs (Supplementary Table S4 and Figure S7) with β-Proteobacteria (22 ± 6%), α-Proteobacteria (20 ± 4%), γ-Proteobacteria (8 ± 2%), and Sphingobacteria (7 ± 2%) dominating the communities. In general, we found relatively little taxa turnover among the various streams, with 97% of the most abundant OTUs (i.e., >1% relative abundance, *n* = 4,900) being present in all 14 sites. More specifically, the 86 most abundant OTUs, accounting for 45.6% of all reads, were detected in all samples. These most abundant OTUs included members of the genera *Rhodoferax* (5 ± 2%), *Novosphingobium* (2 ± 1%), and *Nitrospira* (1 ± 0.4%). Across all streams, we identified 36 obligate methanotrophic OTUs annotated as *Methylococcaceae* (γ-Proteobacteria; i.e., low-affinity, type-I MOB). The relative abundance of methanotrophic OTUs ranged between 0.001% in the alpine stream Adn-1 and 2% in the lowland stream Vey-2 with an average of 0.56 ± 0.58% (Figure 3). Refining their taxonomic assignment revealed that on average 60% of MOB were affiliated with *Crenothrix* spp. across all sites (Figure 4). The most abundant *Crenothrix*-related OTU, OTU222, was consistently detected in all 14 sites and numerically dominated the methanotrophic community in 13 samples. Seven out of the 36 methanotrophic OTUs were detected in 13 out of 14 sites. They include four *Crenothrix* OTUs, OTU222 (relative abundance of 40.5 ± 18.2% of the methanotrophic community fraction), OTU155 (20.2 ± 10.1%), OTU1184 (2.4 ± 2.3%), and OTU2012 (3.5 ± 3.9%); one OTU classified as *Methylobacter* (OTU4584, 6.8 ± 5.1%); and one OTU classified as *Methylomonas* (OTU621, 4.2 ± 2.8%). Across all streams, the relative abundance of *Methylococcaceae* was positively correlated with streamwater CH_4_ concentration (*R*^2^ = 0.85, *p* < 0.01). In turn, *V*_*max*_ was positively related to the relative abundance of *Methylococcaceae* (*R*^2^ = 0.89, *p* < 0.01) as well as to the number of *pmoA*-harboring cells (*R*^2^ = 0.99, *p* < 0.01).

**FIGURE 5:**
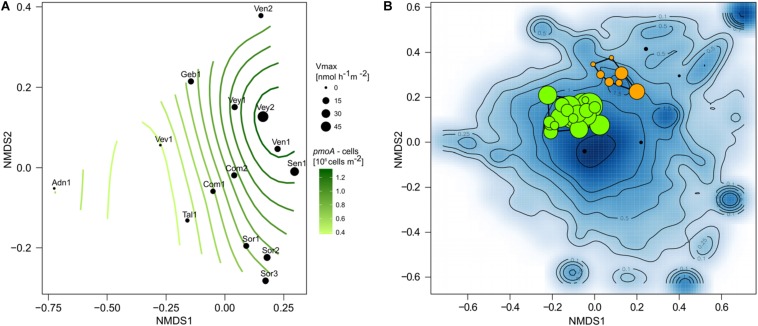
Non-metric multidimensional scaling ordination (nMDS) based on Bray–Curtis similarities of stream sediment communities **(A)**. Maximum reaction velocity (*V*_*max*_) measured at each site is indicated by symbol size, and a GAM model (surface isoclines) represents the distribution of *pmoA* gene counts among communities. Microbial community landscape featuring distinct density peaks of co-occurring operational taxonomic units (OTUs) **(B)**. Shown is the density distribution of species scores (blue background). Isoclines highlight distinct groups of OTUs with similar distribution among the samples. Methanotrophic OTUs (colored circles) mainly fall within two regions and can be grouped in cluster 1 (green) and cluster 2 (orange). Symbol size represents the relative abundance of methanotrophic OTUs.

Community composition shifted along the environmental gradient, and this pattern co-varied significantly with *pmoA* gene counts (log-transformed; *R*^2^ = 0.80, *p* < 0.05) and *V*_*max*_ (*R*^2^ = 0.69, *p* < 0.05; Figure 5A). The microbial composition landscape featured 13 distinct density peaks at a density cutoff of 0.5 (estimated kernel densities ranged from 0 to 6.53). Each of these peaks in the community landscape reflects a group of co-varying OTUs (Figure 5B). Methanotrophic OTUs formed two distinct clusters containing 23 (cluster 1) and seven (cluster 2) OTUs. Six rather low-abundant methanotrophic OTUs were not comprised within these two clusters. Cluster 1 was dominated by the most abundant methanotrophic OTUs *Crenothrix*, *Methylobacter*, and *Methylomonas*. Members of this cluster were found in streams with high CH_4_ concentration. Cluster 2 was dominated by OTUs affiliated with *Methylomonas*, *Methylogaea*, and *Methylosarcina* but also contained low-abundant OTUs classified as *Crenothrix* and *Clonothrix*. Members of cluster 2 were only detected in a subset of streams (Sen-1, Ven-1, Ven-2, Vey-1, and Vey-2), all of which, except Sen-1, were dominated by forests in their catchments (Figure 1A). A convex hull around cluster 1 contained 1,189 non-MOB OTUs, whereas MOB in cluster 2 co-varied with 348 non-methanotrophic OTUs. Both clusters were dominated by unclassified bacterial OTUs (accounting for 21.9 and 67.5% of reads in cluster 1 and cluster 2, respectively), but OTUs classified as non-methanotrophic γ-Proteobacteria (21.2 and 3.2%), δ-Proteobacteria (11.6 and 2.7%), and Acidobacteria (5.5 and 9.4%) also co-varied with the two methanotrophic clusters. In contrast, abundant taxonomic groups such as β-Proteobacteria, α-Proteobacteria, and Bacteroidetes were underrepresented in both methanotrophic clusters (Supplementary Figure S8).

## Discussion

Our study provides insights into the ecophysiology of stream sediment MOB by studying MOX kinetics, MOB community composition, and co-occurrence patterns with non-MOB across a gradient of land cover and associated physicochemical characteristics. Taken together, the results underpin the coupling between CH_4_ supply, MOX kinetics, and MOB structure in streams. Specifically, we found that *V*_*max*_ and *K*_*S*_ increased in streams with elevated CH_4_ supply. *K*_*S*_ values in the μM range matched those measured in shallow streambeds and suggest that low-affinity MOB adapted to high CH_4_ concentrations drive MOX in stream sediments. In line with this, MOB communities were dominated by *Crenothrix*, a type-I MOB belonging to the *Methylococcaceae* family. Our work thus suggests that the attenuation of CH_4_ by MOX scales with CH_4_ availability in streams. CH_4_ availability across these streams was linked to land cover and particularly NO_3_^–^, an indicator of human impact. The ratio of streamwater CH_4_:CO_2_, a proxy for the contribution of methanogenesis to ecosystem metabolism (Stanley et al., 2016) was also elevated in streams with high NO_3_^–^ concentrations. This reflects the potential for *in situ* CH_4_ production linked to oxygen-poor microhabitats in the streambed of streams with elevated NO_3_^–^ concentration and draining catchments dominated by agriculture. As reported earlier (Trimmer et al., 2009, 2010), the ecological niche of stream MOX appears to be restricted to benthic sediments, which agrees with the absence of MOX in streamwater incubations. This is relevant, given the estimated CH_4_ emission of 26.8 Tg CH_4_ per year from streams and rivers (Stanley et al., 2016) and the role of MOX as a sink of CH_4_ at the interface between supersaturated groundwater and streamwater, from which CH_4_ evades to the atmosphere.

**FIGURE 6:**
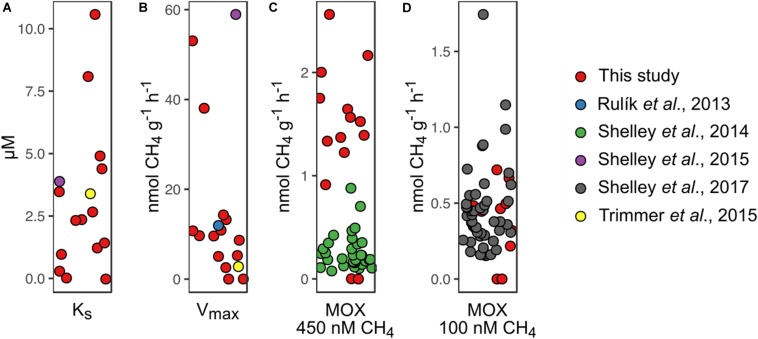
Comparison of methane (CH_4_) oxidation (MOX) rates measured in this study with MOX rates obtained by reanalyzing data from other studies. **(A)** Concentration at half *V*_*max*_ (*K*_*S*_), **(B)** maximum reaction velocity (*V*_*max*_), **(C)** MOX rates at an initial CH_4_ concentration of 450 nM, and **(D)** MOX rates at an initial CH_4_ concentration of 100 nM are shown.

The relationships between the relative abundance of *Methylococcaceae*, *pmoA*-harboring cells, and both *V*_*max*_ and *K*_*S*_ point to the functional relevance of these MOB in streams. Our finding that the potential for MOX is related to *pmoA* copy numbers is consistent with the prevalence of aerobic MOB for the membrane-bound particulate form of CH_4_ monooxygenase (*pMMO*, one subunit being encoded by *pmoA*) as compared to the soluble form (*sMMO*) (Ho et al., 2013; Knief, 2015). Both the relative abundance of *Methylococcaceae* and *pmoA*-harboring cell numbers were related to *V*_*max*_, highlighting the link between MOB abundance and MOX activity (at non-limiting CH_4_ concentrations). In contrast, *K*_*S*_ is independent of the abundance of MOB but depends on the overall affinity of CH_4_ monooxygenases. As substrate affinity exerts a selective force, *K*_*S*_ values are expected to match *in situ* substrate concentrations (Tilman, 1977; Martens-Habbena et al., 2009; Kits et al., 2017). *K*_*S*_ values reported here were on average approximately 50 times greater than streamwater CH_4_ concentrations but in the range of shallow porewater CH_4_ concentrations. This suggests that MOB in shallow stream sediments thrive on elevated CH_4_ concentrations. CH_4_ concentrations increased with sediment depth, a pattern that was noted earlier (Sanders et al., 2007; Rulík et al., 2013; Bednařík et al., 2015; Mach et al., 2015). The interface between oxygen-rich streamwater and CH_4_-enriched porewater may therefore represent a hot spot for MOX. However, given the dominance of *Methylococcaceae* and particularly *Crenothrix* across all sites, the variability of *K*_*S*_ may reflect ecophysiological adaptations of these key players to CH_4_ supply. Since streambed CH_4_ concentrations vary in space and time (Stanley et al., 2016; Crawford et al., 2017; Natchimuthu et al., 2017), flush-feeding on transient CH_4_ pulses may be one of the strategies to maintain methanotrophic activity in stream sediments (Stanley et al., 2016). A comparable phenomenon has been reported for rice paddy soils, where methanotrophs can transiently uphold high-affinity MOX activity (Cai et al., 2016). The fact that MOB communities were dominated by type I MOB which are responsive to changes in CH_4_ concentrations may support this notion. Kinetic information of MOX in streams is scarce, but reanalyzing published data (Shelley et al., 2015; Trimmer et al., 2015) (Supplementary Material), we found our MOX kinetic estimates match those reported from English chalk streams and a lowland stream in the Czech Republic (Figure 6).

Type I *pmoA* (i.e., γ-proteobacterial) is typically expressed by low-affinity MOB in environments with elevated and fluctuating CH_4_ concentrations (Ho et al., 2013). *Methylococcaceae* are such type I MOB (Bowman, 2015), and *K*_*S*_ values in the micromolar range corroborate the low substrate affinity of these MOB. Our results contrast those from chalk streams, where *pmoA* gene sequencing revealed equitable distributions of type I and II MOB (Trimmer et al., 2015). While the presence of type II MOB within the sediments of chalk streams may be attributable to comparatively low CH_4_ concentration, we did not find type II MOB in streams with low CH_4_ supply. However, methanotrophs appear to be rare (i.e., <2% of reads in our 16S rRNA gene survey) and the MOB inventory might not have been fully captured by the 16S rRNA gene sequencing approach. Sequencing of the genes encoding pMMO (*pmoCAB*) might be a more sensitive approach, and future investigations may consider this strategy. Yet, the microbial community landscape analysis revealed two clusters of MOB (Figure 5B)—one containing most of the abundant MOB OTUs and reflecting a shared core community and another cluster of rare MOB, shared only among a subset of streams with forested catchments. There was no consistent taxonomic separation of these clusters. Therefore, whereas a dichotomy between type I and II dominated MOB communities may reflect MOB lifestyle strategies, a more nuanced continuum of trait and taxa distributions appears more likely to underlie the adaptations of MOB to the wide range and variable CH_4_ concentrations within and among streams. For instance, a whole suite of *pmoA*-encoded enzymes may cover a large gradient of CH_4_ affinities (Baani and Liesack, 2008).

Strikingly, we found across all streams only MOB belonging to the *Methylococcaceae* family. Most abundant among the *Methylococcaceae* was *Crenothrix*, accounting for 60% of the reads. *Crenothrix* is a large filamentous γ-Proteobacteria that was originally identified as an aerobic CH_4_ oxidizer in drinking water distribution networks (Völker et al., 1977; Stoecker et al., 2006). *Crenothrix* has recently been reported as a critical component of the methanotrophic communities in temperate lakes (Oswald et al., 2017), a cave ecosystem (Karwautz et al., 2018), and in a eutrophic littoral wetland (Siljanen et al., 2011). The ecological success of *Crenothrix* might be related to its ability to use NO_3_^–^ besides oxygen as an electron acceptor (Oswald et al., 2017). In the microbial community landscape analysis, *Nitrospira*, a nitrite-oxidizing bacterium, is among the most abundant non-methanotrophic OTUs falling within the methanotrophic cluster 1. This co-occurrence may indicate a syntrophic relationship between *Crenothrix* and *Nitrospira* in stream sediments—a hypothesis that requires further investigation. Nevertheless, our study highlights the key role of *Crenothrix* in CH_4_ cycling in freshwater ecosystems.

Methanotrophic carbon fixation can account for up to 46% of benthic phototrophic production (Shelley et al., 2014) and putative carbon fixation rates of up to 13.5 nmol C m^–^^2^ h^−1^ estimated here highlight the functional relevance of MOB at the ecosystem scale. Mediated by diverse interactions with methylotrophs and heterotrophs, MOB may thus significantly contribute to the functioning of stream benthic communities. For instance, MOB were previously shown to sustain diverse communities of non-methanotrophic bacteria in biofilms in a mineral spring cave (Karwautz et al., 2018) and in marine hydrocarbon seeps (Paul et al., 2017). In our study, microbial community structure co-varied with *V*_*max*_ and *pmoA*-harboring cell numbers. Such analyses cannot resolve to which extent this may be due to the role of interactions among microbes or due to shared environmental preferences along the environmental gradient. However, MOB can produce copious amounts of extracellular polymeric substances (EPSs) (Strong et al., 2015; Karwautz et al., 2018), which is understood as an energy-spilling mechanism to prevent the accumulation of toxic formaldehyde. Given the importance of biofilm formation for benthic stream communities (Battin et al., 2016), EPS production may be yet another important role MOB fulfill in stream ecosystems.

In conclusion, this work contributes to our understanding of the ecology of MOB in streams by identifying the coupling between CH_4_ supply and the kinetics of methanotrophic activity. Substrate affinities of a phylogenetically homogeneous MOB population dominated by *Crenothrix*, particularly in lowland streams impacted by agriculture, suggest that the attenuation of CH_4_ by MOX might scale with CH_4_ availability in streams. This is important with respect to the greenhouse gas potential of CH_4_.

## Data Availability Statement

The datasets generated for this study can be found in the NCBI SRA SAMN07174972–SAMN07174985.

## Author Contributions

All authors listed have made a substantial, direct and intellectual contribution to the work, and approved it for publication.

## Conflict of Interest

The authors declare that the research was conducted in the absence of any commercial or financial relationships that could be construed as a potential conflict of interest.
