# Supplementary material for: Microbial Ecology of Methanotrophy in Streams Along a Gradient of CH4 Availability
Source: Front Microbiol. 2020 May 14;11:771. doi: 10.3389/fmicb.2020.00771 (PMC7241049; doi:10.3389/fmicb.2020.00771)
Supplement: Supplementary file 1 [file Data_Sheet_1.pdf]

SUPPLEMENTARY MATERIAL TO

Microbial ecology of methanotrophy in streams along a gradient of CH_4 availability

Alexandre Bagnoud[1,2], Paraskevi Pramateftaki[1], Matthew J. Bogard[3,4], Tom J. Battin[1] and Hannes Peter[1]

[1]Stream Biofilm and Ecosystem Research Laboratory, School of Architecture, Civil and Environmental Engineering, École Polytechnique Fédérale de Lausanne, Lausanne CH-1015, Switzerland

[2]Current address: Haute École d'Ingénierie et de Gestion du Canton de Vaud, Institut de Génie Thermique, CH-1400 Yverdon-les-Bains, Switzerland

[3]Groupe de recherche interuniversitaire en limnologie, Département des sciences biologiques, Université du Québec à Montréal, Montréal, QC, Canada.

[4]Current address: Department of Biological Sciences, University of Lethbridge, Lethbridge, AB, Canada

Supplementary Methods

Calculation of MOX kinetics and oxygen demand

After each sampling, streamwater CH_4, CO_2 and O_2 concentrations in the chambers were calculated as

$$C_a = \frac{C_b \cdot (V_c - V_s) + C_r \cdot V_s}{V_c} \quad (1),$$

where Ca is the expected concentration after sampling, C_b the measured concentration (before sampling), V_c the volume of the chamber, V_s the volume sampled (identical to the replaced volume), and C_r the concentration in the replaced water. Between each sampling time point, the concentrations of O_2, CO_2 and CH_4 were calculated as

$$AC_i = (C_{a,i-1} + C_{b,i})/2 \quad (2),$$

where AC_i is the average concentration at time point i, $C_{a,i-1}$ the concentration after sampling (i.e., accounting for the dilution effect owing to sampling) of previous time point *i*-1, and $C_{b,i}$ the concentration before the sampling (i.e., not accounting for the dilution effect of the sampling) of the time point i. Similarly, the consumption rates of O_2, CO_2 and CH_4 were calculated between each time-point as

$$CR_i = (C_{a,i-1} - C_{b,i})/\Delta t) \quad (3),$$

where CR_i is the consumption at time point, *i*, and Δt the time of *i* and *i*-1, respectively.

Rates of MOX were calculated using Michaelis-Menten kinetics as

$$v = V_{max} \cdot S/(K_s + S) \quad (4),$$

where v is the MOX velocity (or rate), V_{max} the maximum reaction velocity, S the CH_4 concentration, and K_s the concentration for which $v = V_{max}/2$ (half-saturation constant). V_{max} and K_s were estimated for each site from all AC and CR values of triplicate experiments using the 'nls' function from nlstools package (Baty et al., 2015) of R v.3.3.1 (R Development Core Team, 2008). Values of V_{max} were normalized to surface area and expressed as nmol h^{-1} m^{-2} sediment.

Putative *in-situ* rates of MOX were then assessed for each stream using the Michaelis-Menten model (equation 4), knowing both kinetics parameters (V_{max} and K_s) and the *in situ* CH_4 concentration (S).

Re-analysis of published data of MOX rates in stream sediments

Only few studies published kinetics data from MOX measured in stream sediments. Since V_{max} and K_S values were not estimated in these studies, we analysed the available raw data as described above. Data were collected directly from the published plots using the web interface WebPlotDigitizer (Rohatgi, 2015; https://automeris.io/WebPlotDigitizer/).

Only four studies observed Michealis-Menten kinetics of MOX in stream sediments, i.e. a decreasing MOX rate with decreasing CH_4 concentrations (Trimmer et al., 2009, 2010; Shelley et al., 2015, 2015). Unfortunately, nls models with data from Trimmer et al. (2009) and Trimmer et al. (2010) did not converge. However, with data from Trimmer et al. (2015), we obtained a K_S value of 3.4 µM and a V_{max} value of 2.73 nmol g^{-1} h^{-1}. With data from Shelley et al. (2015), we found a K_S value of 3.9 µM and a V_{max} value of 58.9 nmol g^{-1} h^{-1}.

Kinetics parameters estimated here can also be compared to the CH_4 oxidation rates measured by Shelley et al. (2014) in sediments from chalk streams. In this study, rates were assessed by linear decreases of CH_4 in incubation chambers amended with 450 nM CH_4. Knowing the kinetics parameter of our 14 streams, we can calculate CH_4 oxidation rates at this concentration, using equation 4. With such CH_4 concentrations, benthic stream sediments would oxidize CH_4 at rates comprised between 0 to 2.56 nmol g^{-1} h^{-1}, with an average of 1.39 nmol g^{-1} h^{-1}. This fits the rates observed by Shelley et al. (2014), ranging from 0.07 to 0.88 nmol g^{-1} h^{-1}. Similarly, expected rates at a CH_4 concentration of 100 nM (0 to 0.72 nmol g^{-1} h^{-1}) fit the ones measured by Shelley et al. (2017) in chalk streams at this concentration (0.16 to 1.75 nmol g^{-1} h^{-1}).

In the study from Rulík et al. (2013), MOX was expressed as mg CH_4 kg^{-1} sediment dry weight day^{-1}, which can be compared to our measurements (all the other rates were expressed on a sediment volume basis). Since this rate was measured under high CH_4 concentration (50 mL of 100% CH_4 were added to 250-mL serum bottles containing 100 g of fresh sediment), it is very likely that at such concentrations, the rate of CH_4 oxidation was saturated and so this rate corresponds to a V_{max} value. This rate of 11.9 nmol g^{-1} h^{-1} is very close to the average V_{max} rate (12.9 nmol g^{-1} h^{-1}) measured here.

Supplementary References

Baty, F., Ritz, C., Charles, S., Brutsche, M., Flandrois, J.-P., and Delignette-Muller (2015). A Toolbox for nonlinear regression in R: The package nlstools. *J. Stat. Softw.* 66, 1–21.

R Development Core Team (2008). *R: a language and environment for statistical computing*. Vienna, Austria: R Foundation for Statistical Computing Available at: http://www.R-project.org.

Rohatgi, A. (2015). *WebPlotDigitalizer: HTML5 based online tool to extract numerical data from plot images*. Version 4.1 Available at: https://automeris.io/WebPlotDigitizer.

Rulík, M., Mach, V., Brablcová, L., Buriánková, I., Badurová, P., and Gratzová, K. (2013). "Methanogenic system of a small lowland stream Sitka, Czech Republic," in *Biomass Now-Cultivation Utilization* (IntechOpen), 395–426. Available at: http://cdn.intechopen.com/pdfs/44416/InTech-Methanogenic_system_of_a_small_lowland_stream_sitka_czech_republic.pdf [Accessed January 12, 2016].

Shelley, F., Abdullahi, F., Grey, J., and Trimmer, M. (2015). Microbial methane cycling in the bed of a chalk river: oxidation has the potential to match methanogenesis enhanced by warming. *Freshw. Biol.* 60, 150–160. doi:10.1111/fwb.12480.

Shelley, F., Grey, J., and Trimmer, M. (2014). Widespread methanotrophic primary production in lowland chalk rivers. *Proc. R. Soc. Lond. B Biol. Sci.* 281, 20132854. doi:10.1098/rspb.2013.2854.

Shelley, F., Ings, N., Hildrew, A. G., Trimmer, M., and Grey, J. (2017). Bringing methanotrophy in rivers out of the shadows. *Limnol. Oceanogr.* 62, 2345–2359. doi:10.1002/lno.10569.

Trimmer, M., Hildrew, A. G., Jackson, M. C., Pretty, J. L., and Grey, J. (2009). Evidence for the role of methane-derived carbon in a free-flowing, lowland river food web. *Limnol. Oceanogr.* 54, 1541–1547. doi:10.4319/lo.2009.54.5.1541.

Trimmer, M., Maanoja, S., Hildrew, A. G., Pretty, J. L., and Grey, J. (2010). Potential carbon fixation via methane oxidation in well-oxygenated river bed gravels. *Limnol. Oceanogr.* 55, 560–568. doi:10.4319/lo.2010.55.2.0560.

Supplementary Table S1. Percent land cover for the catchments of the 14 sites estimated from the 2018 Corine land cover database.

	Non-irrigated arable land	Mixed forest	Coniferous forest	Natural grasslands	Bare rocks	Discontinuous urban fabric	Land principally occupied by agriculture	Sparsely vegetated areas	Transitional woodland-shrub	Broad-leaved forest	Pastures	Moors and heathland	Industrial or commercial units	Glaciers and perpetual snow
Adn-1	0.0	4.4	6.3	19.3	52.3	0.0	0.0	12.1	1.6	0.0	0.0	2.6	0.0	1.3
Com-1	96.9	3.0	0.0	0.0	0.0	0.1	0.0	0.0	0.0	0.0	0.0	0.0	0.0	0.0
Com-1	96.9	3.0	0.0	0.0	0.0	0.1	0.0	0.0	0.0	0.0	0.0	0.0	0.0	0.0
Geb-1	96.8	3.2	0.0	0.0	0.0	0.1	0.0	0.0	0.0	0.0	0.0	0.0	0.0	0.0
Sen-1	96.5	3.1	0.0	0.0	0.0	0.4	0.0	0.0	0.0	0.0	0.0	0.0	0.0	0.0
Sor-1	92.8	1.0	0.0	0.0	0.0	5.1	0.0	0.0	0.0	0.0	0.0	0.0	0.9	0.0
Sor-2	92.8	1.0	0.0	0.0	0.0	5.1	0.0	0.0	0.0	0.0	0.0	0.0	0.9	0.0
Sor-3	92.8	1.0	0.0	0.0	0.0	5.1	0.0	0.0	0.0	0.0	0.0	0.0	0.9	0.0
Tal-1	86.9	6.7	0.0	0.0	0.0	6.0	0.0	0.0	0.0	0.0	0.4	0.0	0.0	0.0
Ven-1	51.9	20.2	13.1	1.5	0.0	5.3	1.2	0.0	1.1	3.2	1.5	0.0	0.6	0.0
Ven-2	53.5	20.9	13.6	1.6	0.0	2.7	1.2	0.0	1.2	3.3	1.6	0.0	0.3	0.0
Vev-1	0.0	6.3	25.9	52.4	0.0	1.5	12.6	0.0	1.2	0.0	0.0	0.0	0.0	0.0
Vey-1	63.7	15.4	15.8	1.9	0.0	0.4	0.8	0.0	1.4	0.3	0.0	0.0	0.0	0.0
Vey-2	65.2	15.3	16.2	1.7	0.0	0.1	0.0	0.0	1.4	0.0	0.0	0.0	0.0	0.0

Supplementary Table S2. Names, localizations and sampling dates of the 14 streams sampled during this survey, together with selected chemical parameters of stream waters.

Stream name	Tag name	GPS (WGS84, °)	Altitude (m)	Sampling date	CH_4 (µM)	O_2 (µM)	CO_2 (µM)	DOC (µM)	Cl^- (mM)	SO_4^{2-} (mM)	NO_3^- (mM)	Ca^{2+} (mM)	pH	Temp. (°C)
La Veveyse	Vev-1	N 46.527289, E 6.922661	868	11.04.2016	0.005	367.19	34.83	133.68	0.044	0.067	0.007	1.032	8.39	5.1
L'Avançon de Nant	Adn-1	N 46.253852, E 7.109585	1189	28.03.2016	0.004	363.33	27.14	14.19	0.004	0.166	0.009	0.724	7.81	2.7
Le Talent	Tal-1	N 46.590450, E 6.666399	723	04.04.2016	0.006	361.09	31.76	156.20	0.195	0.117	0.050	1.020	8.22	7.4
Le Combagno[illegible]	Com-1	N 46.574373, E 6.441875	594	02.05.2016	0.011	346.77	72.09	320.11	0.180	0.125	0.125	1.928	8.38	9.3
Le Veyron	Vey-1	N 46.640305, E 6.483072	523	17.05.2016	0.018	338.54	62.92	202.48	0.189	0.086	0.126	1.134	7.76	9.5
Le Veyron	Vey-2	N 46.584923, E 6.400136	663	28.06.2016	0.572	248.75	197.95	222.31	0.182	0.087	0.147	1.953	7.93	11.5
La Sorge	Sor-1	N 46.522506, E 6.571378	386	14.03.2016	0.134	369.69	37.74	124.24	0.434	0.446	0.150	1.379	8.20	5.9
Le Combagno[illegible]	Com-2	N 46.574073, E 6.441998	594	02.05.2016	0.053	339.58	71.82	328.37	0.317	0.121	0.182	2.081	8.37	5.9
La Venoge	Ven-1	N 46.525409, E 6.545010	378	21.03.2016	0.120	338.13	82.56	149.04	0.506	0.231	0.248	1.361	7.96	7.4
La Sorge	Sor-3	N 46.522506, E 6.571378	386	20.06.2016	0.124	306.56	60.81	242.70	0.479	0.359	0.254	1.682	8.30	12.8
La Venoge	Ven-2	N 46.551325, E 6.540395	391	04.07.2016	0.234	299.38	85.34	144.90	0.429	0.318	0.256	2.507	8.19	15.7
La Gèbre	Geb-1	N 46.624060, E 6.462538	575	11.07.2016	0.125	225.63	306.62	204.41	0.316	0.229	0.260	2.631	7.97	15.6
La Sorge	Sor-2	N 46.522506, E 6.571378	386	14.06.2016	0.133	290.63	51.69	247.11	0.477	0.353	0.263	1.753	8.40	13.9
La Senoge	Sen-1	N 46.556722, E 6.482098	489	25.04.2016	0.336	315.63	142.43	206.06	0.613	0.229	0.372	1.959	8.16	8.6

Supplementary Table S3. Methanotrophic community contribution, V_{max} and K_S values of MOX incubations, CH_4 concentrations in stream- and shallow porewaters, computed *in situ* rates of MOX and rates of methanotrophic carbon fixation for all fourteen streams.

			Methanotrophs			CH_4 oxidation kinetics			CH_4 concentrations in streams			*In-situ* rates			
Stream	Sediment surface area	Microbial cells density	abundance of Methylo-coccaceae	*pmoA*-harboring cells density		Vmax		Ks	CH_4 conc. in stream-water	CH_4 conc. in porewater	CH_4 conc. 2.5 cm below streambed (interpolated)	Putative *in-situ* CH_4 oxidation rate		Putative *in-situ* carbon fixation rate by methanotrophs	
	(m^2 g^{-1})	(cells m^{-2})	(%)	(cells m^{-2})	(%)	(nmol CH_4 g^{-1} h^{-1})	(nmol CH_4 m^{-2} h^{-1})	(µM)	(µM)	(µM)	(µM)	(nmol CH_4 g^{-1} h^{-1})	(nmol CH_4 m^{-2} h^{-1})	(nmol C g^{-1} h^{-1})	(nmol C m^{-2} h^{-1})
Adn-1	1.362	1.91×10^7	0.001	< d. l.	0.00%	0	0	0	0.004	0.011 (at 8 cm)	0.006	0	0	0	0
Com-1	0.856	3.62×10^8	0.061	5.5×10^5	0.15%	5.25	6.13	0.98	0.011	1.493 (at 9.7 cm)	0.394	2.62	3.07	1.31	1.53
Com-2	0.999	3.24×10^8	0.255	5.92×10^5	0.18%	9.68	9.69	2.68	0.053	0.503 (at 9 cm)	0.178	4.84	4.85	2.42	2.42
Geb-1	0.981	1.69×10^8	0.118	9.66×10^5	0.57%	9.54	9.72	1.23	0.125	65.75 (at 5 cm)	32.935	4.77	4.86	2.38	2.43
Sen-1	1.263	8.22×10^7	1.334	2.18×10^6	2.65%	38.06	30.13	8.11	0.327	201.9 (at 10 cm)	50.735	19.03	15.06	9.52	7.53
Sor-1	0.992	5.62×10^7	0.460	5.94×10^5	1.06%	8.61	8.68	2.38	0.134	6.859 (at 10 cm)	1.815	4.31	4.34	2.15	2.17
Sor-2	0.863	7.86×10^7	0.499	8.3×10^5	1.06%	13.26	15.36	3.47	0.133	101.8 (at 9.3 cm)	27.354	6.63	7.68	3.31	3.84
Sor-3	0.886	1.85×10^8	0.393	7.33×10^5	0.40%	10.69	12.07	2.30	0.124	5.159 (at 10 cm)	1.383	5.34	6.03	2.67	3.02
Tal-1	1.154	5.35×10^7	0.063	1.39×10^5	0.26%	2.55	2.21	0.28	0.006	0.847 (at 10 cm)	0.216	1.27	1.10	0.64	0.55
Ven-1	1.365	5.22×10^7	0.952	4.95×10^5	0.95%	14.31	10.48	4.37	0.120	0.008 (at 10 cm)	0.092	7.16	5.24	3.58	2.62
Ven-2	0.982	9.79×10^7	0.586	5.65×10^5	0.58%	5.03	5.12	1.40	0.234	47.70 (at 10 cm)	12.101	2.51	2.56	1.26	1.28
Vev-1	5.501	4.6×10^6	0.076	8.41×10^3	0.18%	0	0	0	0.005	0.024 (at 6 cm)	0.013	0	0	0	0
Vey-1	1.012	1.90×10^8	0.986	8×10^5	0.42%	10.91	10.78	4.92	0.018	4.821 (at 8.7 cm)	1.403	5.46	5.39	2.73	2.69
Vey-2	0.986	2.81×10^8	1.996	3.77×10^6	1.34%	53.09	53.86	10.59	0.572	9.072 (at 6 cm)	4.114	26.55	26.93	13.27	13.47

Supplementary Table S4. OTU table of the 16S rRNA sequencing analysis, including the taxonomic annotation and the sequence of each OTU (online).

Supplementary Figures

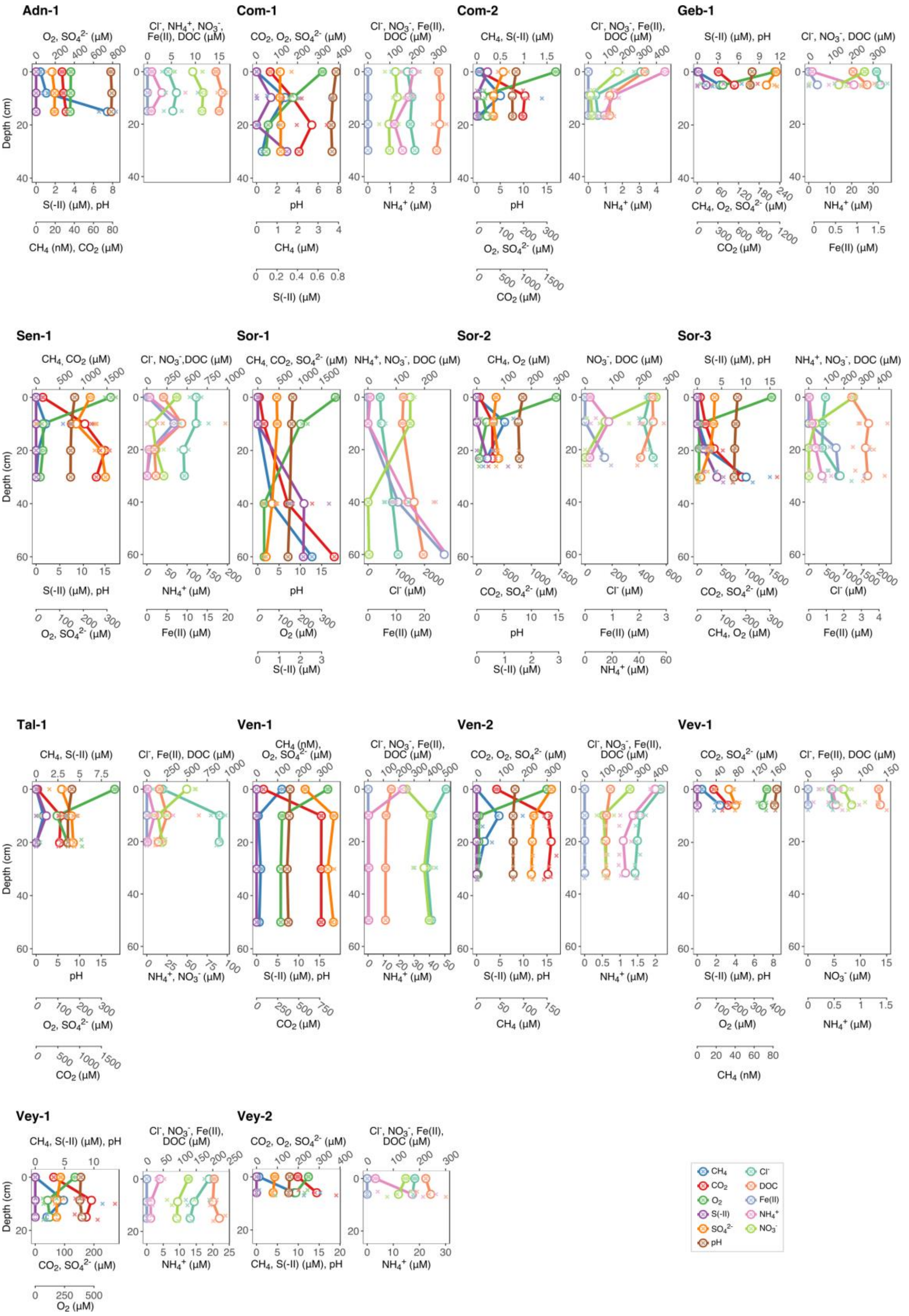

Supplementary Figure S1. Stream porewater profiles. Open symbols represent replicates, plain symbols represent averaged values.

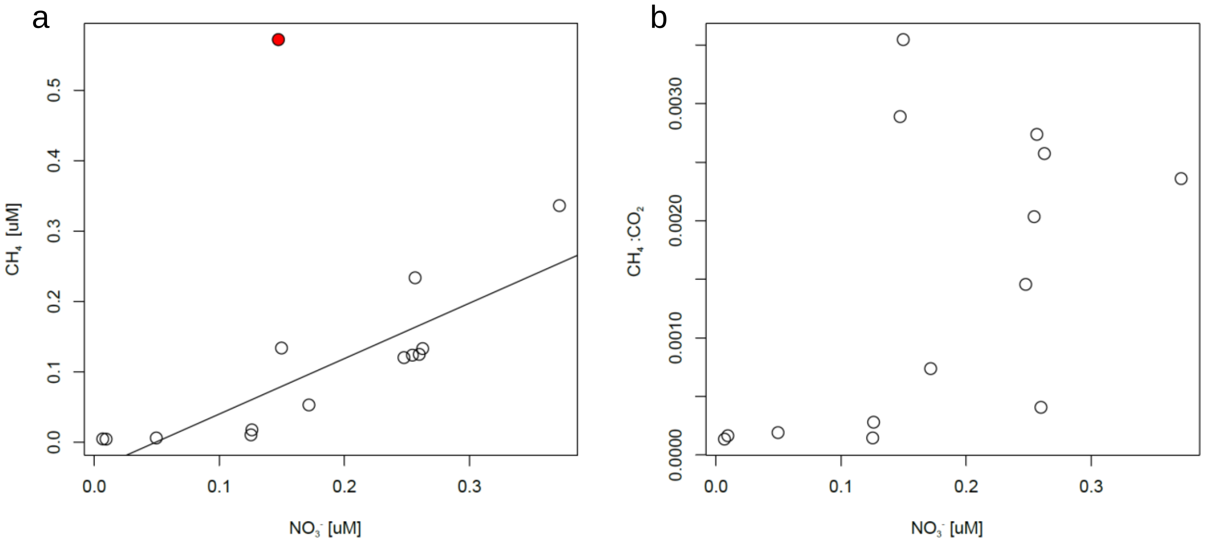

Supplementary Figure S2. Streamwater CH_4 concentration was significantly positively related to NO_3^- concentration (a) when site Vey-2 (in red) is excluded (see main text). Also, the ratio of streamwater CH_4 to CO_2 increased in streams with elevated NO_3^- concentrations (b).

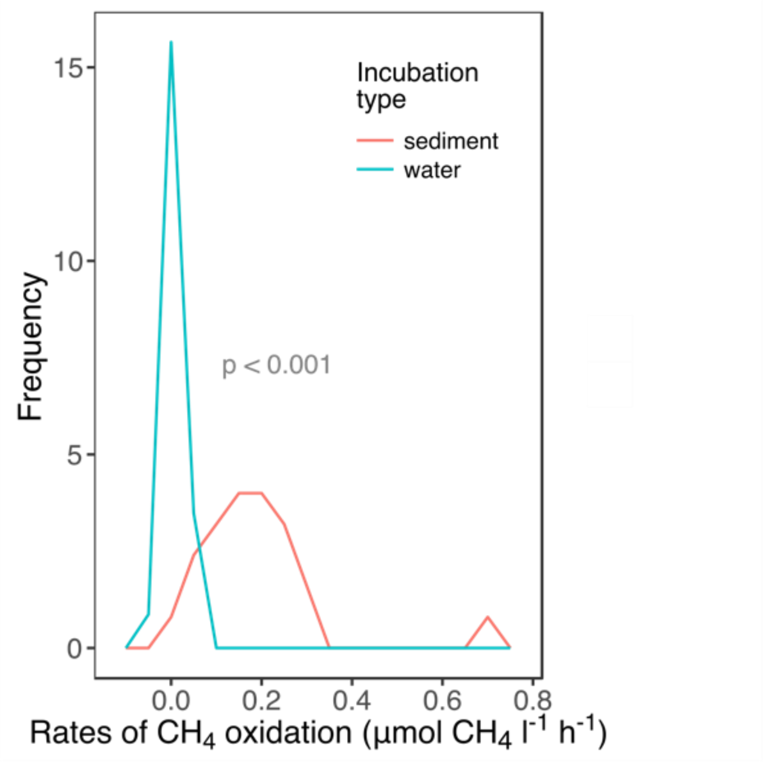

Supplementary Figure S3. Frequency distribution of MOX rates measured in laboratory incubations during the first 24 h. The blue curve represents the incubations with streamwater only, while the red curve represents incubations with sediments (in streamwater). Incubations with sediments had significantly higher MOX rates than incubations without (ANOVA, $p < 0.001$), suggesting that methanotrophic activity mainly takes place in streambed sediments.

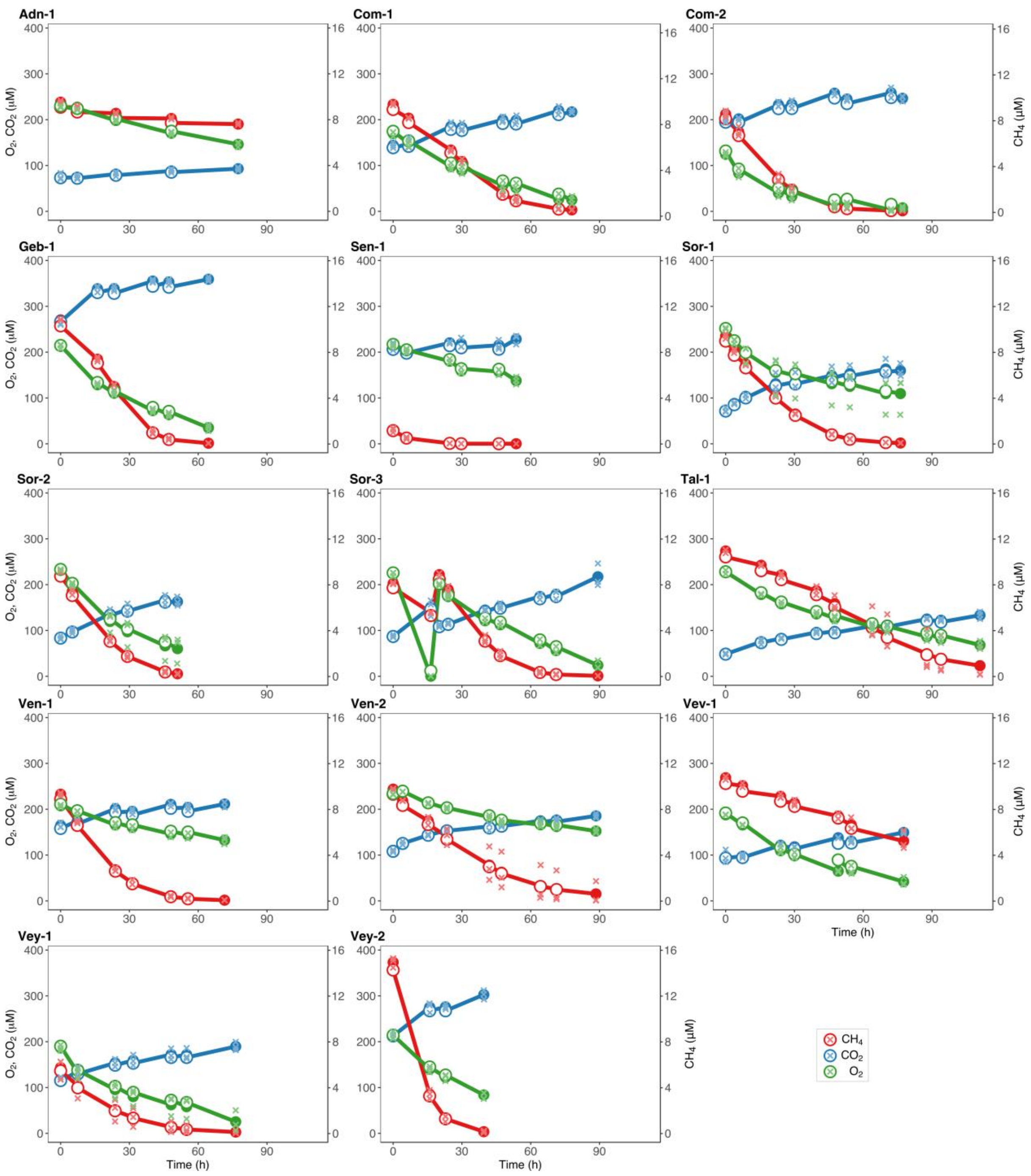

Supplementary Figure S4. Change over time of CH_4 (red symbols), CO_2 (blue symbols) and O_2 (green symbols) in laboratory incubations of ~ 25 g of sediment in stream water amended with CH_4. Crosses represent replicates, filled circles represent averages, and open circles represent average concentration accounting for the dilution due to sampling. The steps occur due to the replacement of water during sampling and is accounted for in the rate measurements.

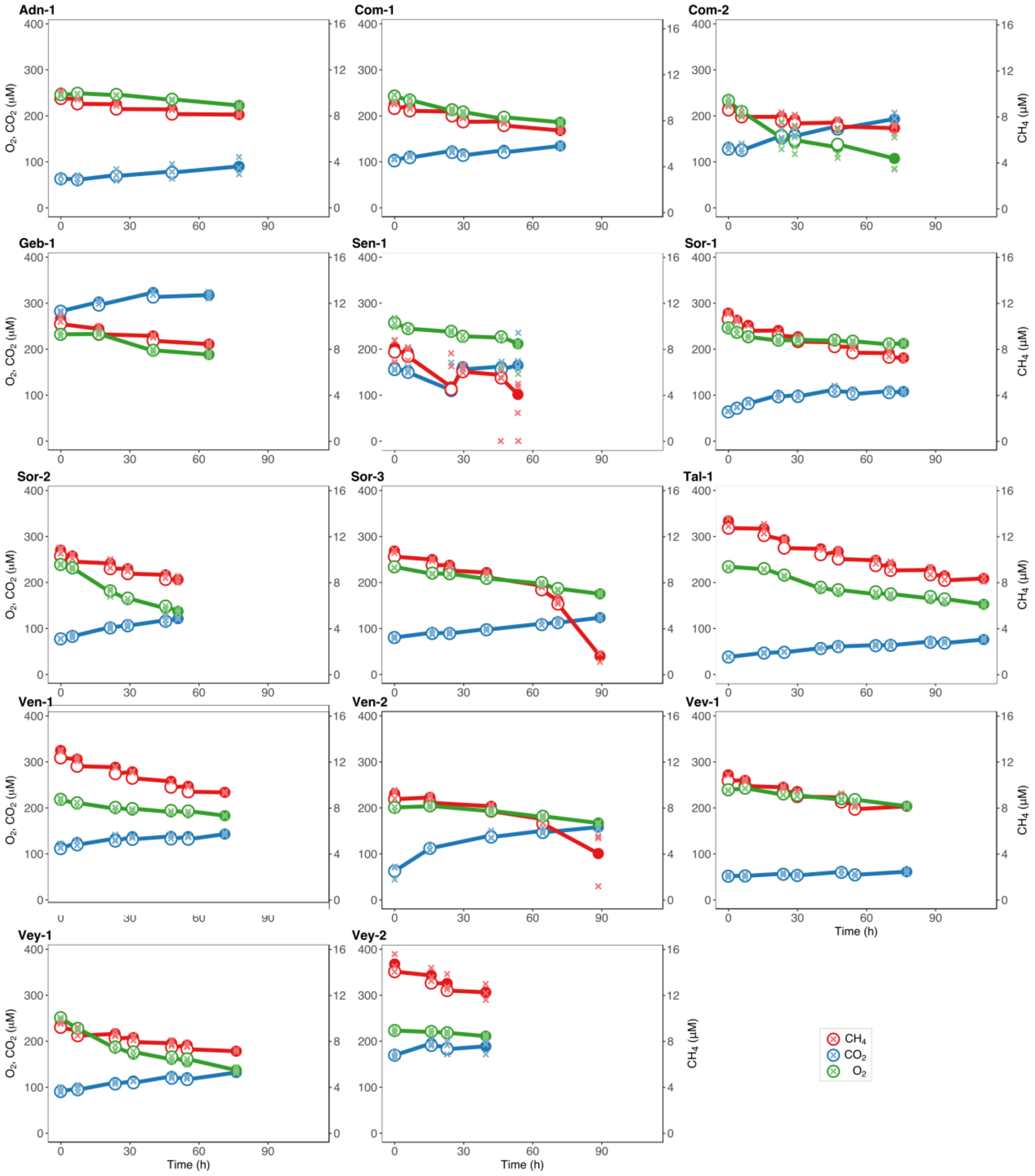

Supplementary Figure S5. Change over time of CH_4 (red symbols), CO_2 (blue symbols) and O_2 (green symbols) in laboratory incubations of stream water only amended with CH_4. Crosses represent replicates, filled circles represent averages, and open circles represent average concentration accounting for the dilution due to sampling. The steps occur due to the replacement of water during sampling and is accounted for in the rate measurements.

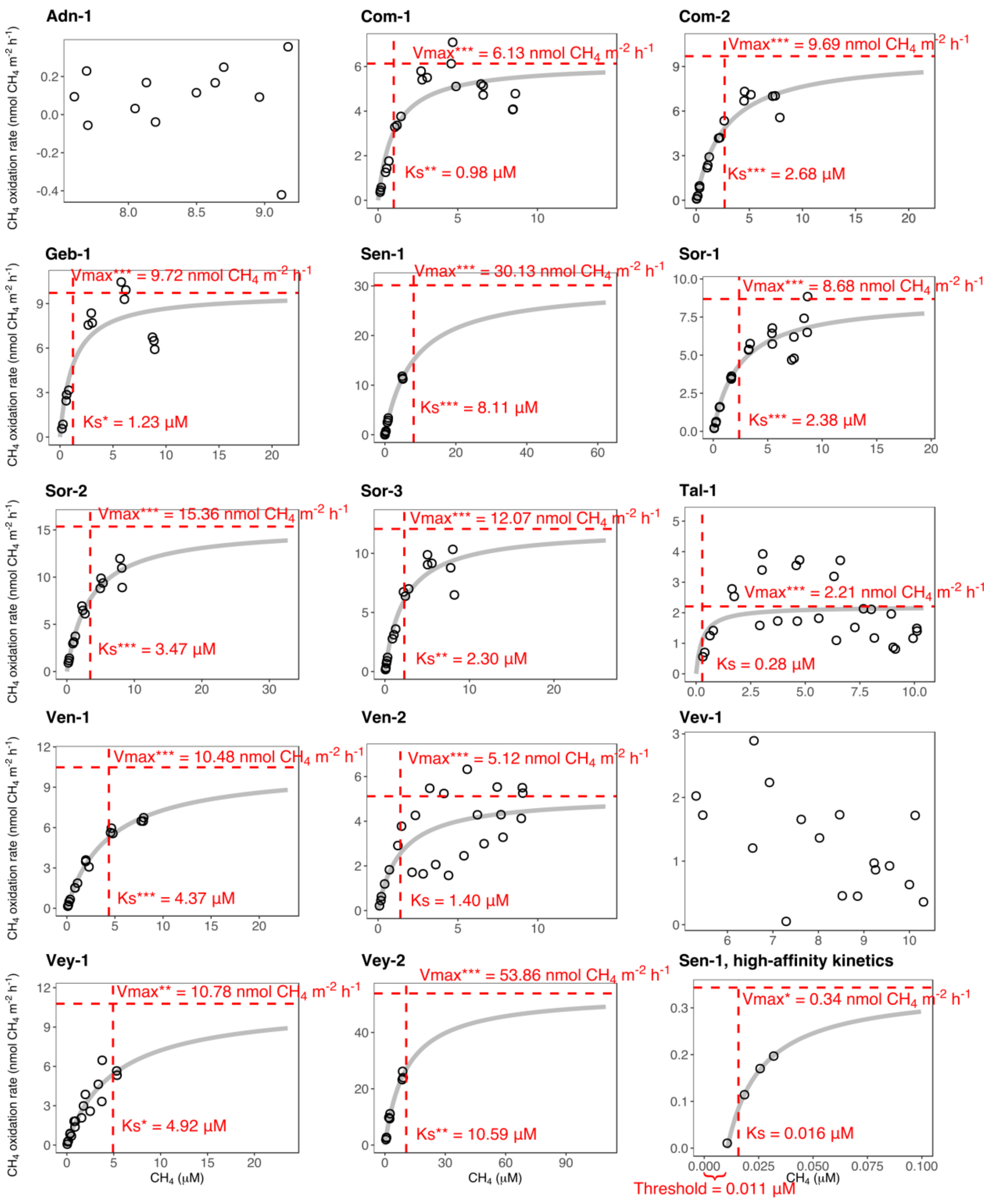

Supplementary Figure S6. Michaelis-Menten models of MOX rates measured in laboratory incubations. Dots represent measurements, grey lines show the kinetic models, vertical and horizontal red dashed lines represent V_{max} and K_S values, respectively. For streams Adn-1 and Vev-1, the kinetics could not be modelled, due to the absence of activity. The p-value of the model's parameters, V_{max} and K_S, is indicated by stars (*** < 0.001 < ** < 0.01 < * < 0.05).

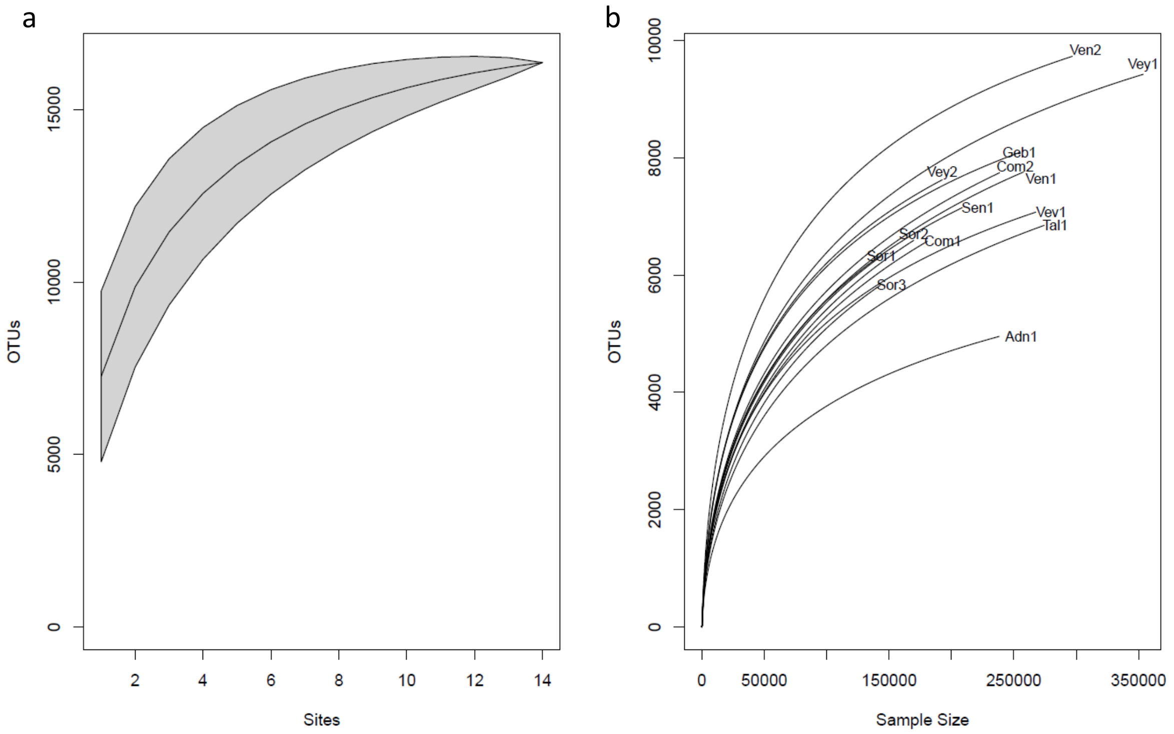

Supplementary Figure S7. Accumulation curve (a) and sample rarefaction curves (b) showing the number of OTUs detected at each site.

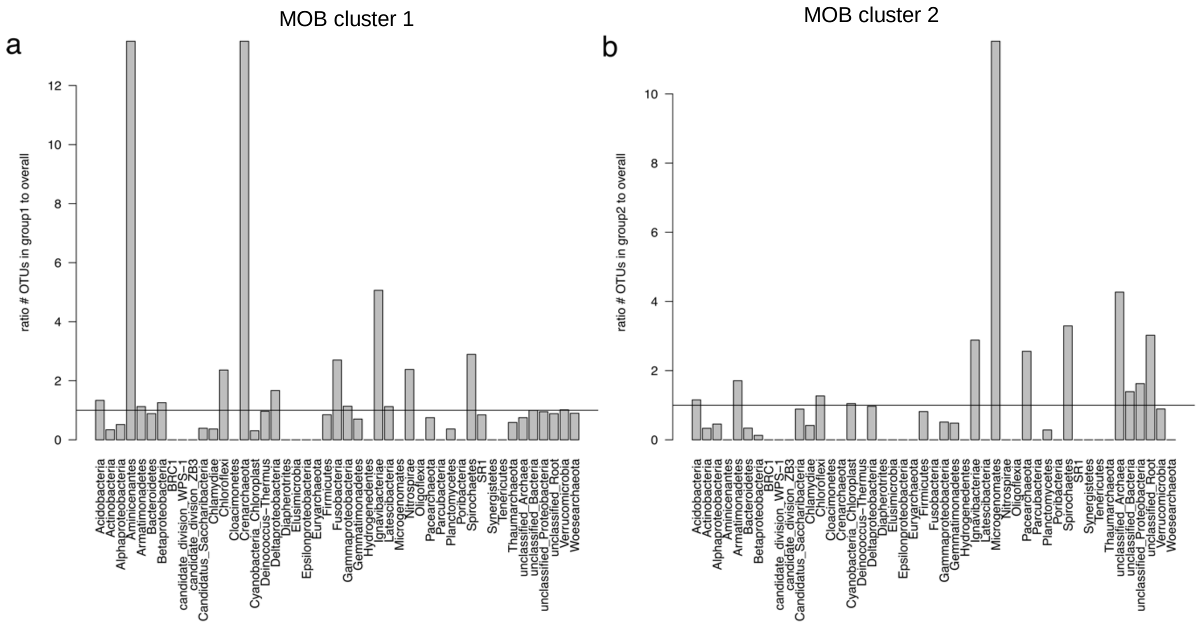

Supplementary Figure S8. Relative abundance ratios of major taxonomic groups co-clustering with MOB cluster 1 (**a**) and MOB cluster 2 (**b**) (as defined by the community landscape analysis). For each taxonomic group, the ratio of relative abundance of OTUs co-clustering with MOBs to the overall relative abundances of the respective groups is shown. Values > 1 (horizontal line) indicate an overrepresentation of taxa co-clustering with MOB cluster 1 and cluster 2 respectively, while values smaller than 1 indicate underrepresentation.
